# Supplementary material for: Microaxial Left Ventricular Assist Device in Cardiogenic Shock: A Systematic Review and Meta-Analysis
Source: Life (Basel). 2022 Oct 18;12(10):1629. doi: 10.3390/life12101629 (PMC9605512; doi:10.3390/life12101629)
Supplement: Supplementary file 1 [file life-12-01629-s001.zip › life-1973239-supplementary.pdf]

Microaxial Left Ventricular Assist Device in Cardiogenic Shock: A Systematic Review and Meta-Analysis

Supplementary Appendix

Shien Ru Tan, MBBS (ORCID: 0000-0001-5144-9930)
Christopher Jer Wei Low, MBBS (ORCID:
Wei Lin Ng[1], MBBS (ORCID: 0000-0002-6932-5458)
Ryan Ruiyang Ling, MBBS (ORCID: 0000-0002-8335-7640),
Chuen Seng Tan, PhD (ORCID: 0000-0002-6513-2309),
Shir Lynn Lim, MMed, MRCP (ORCID: 0000-0002-1151-2357)
Robin Cherian, MRCP
Weiqin Lin, MRCP
Kiran Shekar, PhD
Saikat Mitra, MD (ORCID: 0000-0002-0525-892X),
Graeme MacLaren, MSc, FCICM (ORCID 0000-0002-1307-4274)
Kollengode Ramanathan, MD, FCICM (ORCID: 0000-0003-1822-9455)

Corresponding author:
Kollengode Ramanathan
Cardiothoracic Intensive Care Unit, National University Heart Centre, National University Hospital, Singapore.
Level 9, 1E Kent Ridge Road, SINGAPORE 119228.
Tel: +6567727862
Email: surrkr@nus.edu.sg
ORCID: 0000-0003-1822-9455

Table of contents

Table S1. Preferred Reporting Items for Systematic reviews and Meta-analyses checklist 3

Table S2. Search strategies for databases 5

Table S3. Definitions of complications reported while patients were receiving microaxial LVAD support. 5

Table S4. American Society of Echocardiography Guidelines on severity of cardiogenic shock based on 2-dimensional echocardiogram-derived left ventricular ejection fraction. 5

Table S5. Results of subgroup analysis 6

Table S6. Results of meta-regression analysis 6

Table S7. Joanna Briggs Institute Critical Appraisal Checklist. 7

Table S8. Grading of Recommendations, Assessment, Development, and Evaluations. 10

References 12

Table S1. Preferred Reporting Items for Systematic reviews and Meta-analyses checklist

Section and Topic	Item #	Checklist item	Location where item is reported
**TITLE**			
Title	1	Identify the report as a systematic review.	Page 1
**ABSTRACT**			
Abstract	2	See the PRISMA 2020 for Abstracts checklist.	Page 2
**INTRODUCTION**			
Rationale	3	Describe the rationale for the review in the context of existing knowledge.	Page 3
Objectives	4	Provide an explicit statement of the objective(s) or question(s) the review addresses.	Page 3
**METHODS**			
Eligibility criteria	5	Specify the inclusion and exclusion criteria for the review and how studies were grouped for the syntheses.	Page 4
Information sources	6	Specify all databases, registers, websites, organisations, reference lists and other sources searched or consulted to identify studies. Specify the date when each source was last searched or consulted.	Page 4
Search strategy	7	Present the full search strategies for all databases, registers and websites, including any filters and limits used.	Page 4, Supplementary Material
Selection process	8	Specify the methods used to decide whether a study met the inclusion criteria of the review, including how many reviewers screened each record and each report retrieved, whether they worked independently, and if applicable, details of automation tools used in the process.	Page 4-5
Data collection process	9	Specify the methods used to collect data from reports, including how many reviewers collected data from each report, whether they worked independently, any processes for obtaining or confirming data from study investigators, and if applicable, details of automation tools used in the process.	Page 4-5
Data items	10a	List and define all outcomes for which data were sought. Specify whether all results that were compatible with each outcome domain in each study were sought (e.g. for all measures, time points, analyses), and if not, the methods used to decide which results to collect.	Page 4-5
	10b	List and define all other variables for which data were sought (e.g. participant and intervention characteristics, funding sources). Describe any assumptions made about any missing or unclear information.	Page 5-6
Study risk of bias assessment	11	Specify the methods used to assess risk of bias in the included studies, including details of the tool(s) used, how many reviewers assessed each study and whether they worked independently, and if applicable, details of automation tools used in the process.	Page 5
Effect measures	12	Specify for each outcome the effect measure(s) (e.g. risk ratio, mean difference) used in the synthesis or presentation of results.	Page 5-6
Synthesis methods	13a	Describe the processes used to decide which studies were eligible for each synthesis (e.g. tabulating the study intervention characteristics and comparing against the planned groups for each synthesis (item #5)).	Page 4-6
	13b	Describe any methods required to prepare the data for presentation or synthesis, such as handling of missing summary statistics, or data conversions.	Page 4-6
	13c	Describe any methods used to tabulate or visually display results of individual studies and syntheses.	Page 4-6
	13d	Describe any methods used to synthesize results and provide a rationale for the choice(s). If meta-analysis was performed, describe the model(s), method(s) to identify the presence and extent of statistical heterogeneity, and software package(s) used.	Page 4-6
	13e	Describe any methods used to explore possible causes of heterogeneity among study results (e.g. subgroup analysis, meta-regression).	Page 4-6
	13f	Describe any sensitivity analyses conducted to assess robustness of the synthesized results.	Page 4-6
Reporting bias assessment	14	Describe any methods used to assess risk of bias due to missing results in a synthesis (arising from reporting biases).	Page 4-6
Certainty assessment	15	Describe any methods used to assess certainty (or confidence) in the body of evidence for an outcome.	Page 4-6
**RESULTS**			

Section and Topic	Item #	Checklist item	Location where item is reported
Study selection	16a	Describe the results of the search and selection process, from the number of records identified in the search to the number of studies included in the review, ideally using a flow diagram.	Page 6, Figure 1
	16b	Cite studies that might appear to meet the inclusion criteria, but which were excluded, and explain why they were excluded.	Page 6, Figure 1
Study characteristics	17	Cite each included study and present its characteristics.	Pages 6-7, Table 1
Risk of bias in studies	18	Present assessments of risk of bias for each included study.	Page 10, Table S7-S8
Results of individual studies	19	For all outcomes, present, for each study: (a) summary statistics for each group (where appropriate) and (b) an effect estimate and its precision (e.g. confidence/credible interval), ideally using structured tables or plots.	Pages 7-9, Figures 2-3, Tables S5-S6
Results of syntheses	20a	For each synthesis, briefly summarise the characteristics and risk of bias among contributing studies.	Page 11, Table S7-S8
	20b	Present results of all statistical syntheses conducted. If meta-analysis was done, present for each the summary estimate and its precision (e.g. confidence/credible interval) and measures of statistical heterogeneity. If comparing groups, describe the direction of the effect.	Page 7-8, Tables S5-S6
	20c	Present results of all investigations of possible causes of heterogeneity among study results.	Page 10, Table S8
	20d	Present results of all sensitivity analyses conducted to assess the robustness of the synthesized results.	Page 7
Reporting biases	21	Present assessments of risk of bias due to missing results (arising from reporting biases) for each synthesis assessed.	Page 10, Table S7
Certainty of evidence	22	Present assessments of certainty (or confidence) in the body of evidence for each outcome assessed.	Figures 2-4, Supplementary Material
**DISCUSSION**			
Discussion	23a	Provide a general interpretation of the results in the context of other evidence.	Pages 10-13
	23b	Discuss any limitations of the evidence included in the review.	Page 13
	23c	Discuss any limitations of the review processes used.	Page 13
	23d	Discuss implications of the results for practice, policy, and future research.	Page 13
**OTHER INFORMATION**			
Registration and protocol	24a	Provide registration information for the review, including register name and registration number, or state that the review was not registered.	Page 4
	24b	Indicate where the review protocol can be accessed, or state that a protocol was not prepared.	Page 4
	24c	Describe and explain any amendments to information provided at registration or in the protocol.	Page 4
Support	25	Describe sources of financial or non-financial support for the review, and the role of the funders or sponsors in the review.	Not applicable
Competing interests	26	Declare any competing interests of review authors.	Page 1
Availability of data, code and other materials	27	Report which of the following are publicly available and where they can be found: template data collection forms; data extracted from included studies; data used for all analyses; analytic code; any other materials used in the review.	Page 1

Table S2. Search strategies for databases

Database	Search strategy
Pubmed	(impella*[tiab]) AND ("Shock, Cardiogenic"[Mesh] OR "cardiogenic shock"[tiab])
Embase	('impella*' OR 'impella':ti,ab) AND ('cardiogenic shock'/exp OR 'cardiogenic shock':ti,ab)
Scopus	TITLE-ABS-KEY (impella* AND (‘cardiogenic shock’))

Table S3. Definitions of complications reported while patients were receiving microaxial LVAD support.

Event	Definition
**Device malfunction**	Kinking of device, device migration or device failure regardless of etiology requiring extraction or replacement of the device.[1]
**Access-site bleeding**	Any of the following:<br>• Bleeding with associated serum hemoglobin level decrease of at least 5 g/dL (=3.1 mmol/L). The decrease in hemoglobin will be calculated as the last recorded Hb measurement preceding the onset of the bleeding, subtracted by the nadir Hb measurement (associated with the bleeding) [1,2]<br>• Bleeding requiring blood transfusions(only blood transfusions that are explicitly related to the bleeding are taken into account) [1-3]<br>• The need for surgery to control the bleeding[1-3]
**Hemolysis**	Either:<br>• Retrospectively diagnosed by Hb decrease and/or the need of blood transfusion, plus either increase in LDH or decrease in haptoglobin.[3,4]<br>• Evidence of clinically relevant hemolysis requiring extraction of device or blood transfusion[2]
**Limb ischaemia**	Hypoperfusion of the leg requiring treatment and marked by such symptoms as decreased skin temperature of the limb or decreased peripheral pulses[5]
**Stroke**	Ischemic or hemorrhagic cerebrovascular accident that persisted beyond 24 hours or less than 24 hours with evidence on imaging study[5]

Table S4. American Society of Echocardiography Guidelines on severity of cardiogenic shock based on 2-dimensional echocardiogram-derived left ventricular ejection fraction [6].

	Male				Female			
	**Normal range**	**Mildly abnormal**	**Moderately abnormal**	**Severely abnormal**	**Normal range**	**Mildly abnormal**	**Moderately abnormal**	**Severely abnormal**
**LVEF (%)**	52–72	41–51	30–40	<30	54–74	41–53	30–40	<30

Table S5. Results of subgroup analysis

	Subgroup	Studies	Mortality(%)	95% CI
Geographical Location (*p*=0.17)	North America	25	41.8	35.6-48.1
	Europe	34	49.1	44.3-54.0
	Asia	3	49.5	34.0-65.2
	South America	1	64.3	37.1-87.7
Pre-device LVEF (p=0.22)	≤20%	11	39.1	30.0-48.6
	>20%	22	46.4	39.5-53.4
Etiologies of CS (p=0.06)	AMICS	13	52.5	46.9-58.1
	NMICS	4	41.2	31.2-41.5
	Mixed (both AMICS and NMICS)	38	44.6	39.0-50.2
Insertion mode (*p*=0.07)	Percutaneous	16	52.4	43.8-61.0
	Surgical	16	40.7	31.6-50.3
Cannulation Access (*p*=0.40)	Axillary Artery	6	47.9	37.9-57.9
	Femoral Artery	15	52.7	47.2-58.3
Duration of device Support (*p*=0.46)	Support ≤ 4 days	17	46.9	40.2-53.8
	Support > 4 days	20	43.7	38.4-49.0
Concomitant ECMO use (*p*=0.04)	Concomitant ECMO	8	51.5	47.1-55.9
	Microaxial LVAD only	40	44.6	39.6-49.6

Abbreviations: AMICS: Acute myocardial infarction cardiogenic shock CI: confidence interval, CS: cardiogenic shock, ECMO: extracorporeal membrane oxygenation, LVEF: left ventricular ejection fraction, NMICS: Non-myocardial infarction cardiogenic shock

Table S6. Results of meta-regression analysis

Covariate	Studies	Estimate	LCI	UCI	P
Smoking	30	-0.0344	-0.2574	0.1886	0.76
CVA	27	0.2852	0.0153	0.5551	**0.038**
AMI	26	0.0831	-0.1573	0.3235	0.50
Age	62	0.0026	-0.0045	0.0097	0.47
Duration of device support [days]	43	-0.0154	-0.0223	-0.0085	**<0.0001**
LVEF	33	0.0053	-0.0012	0.0118	0.11
CHF	23	0.0893	-0.0682	0.2467	0.27
Male gender	56	0.1457	-0.1093	0.4006	0.26
HLD	34	0.675	0.0162	0.3187	**0.030**
DM	47	0.0402	-0.3353	0.4156	0.83
HTN	41	0.0739	-0.1230	0.2709	0.46

Abbreviations: AMI: acute myocarditla infarction, CHF: Congestive heart failure, CVA: cerebrovascular accident, DM: diabetes mellitus ,HLD: hyperlipidemia, HTN: hypertension, LCI: lower confidence interval, LVEF: left ventricular ejection fraction, UCI: upper confidence interval
*Rows in bold: p<0.05.

Table S7. Joanna Briggs Institute Critical Appraisal Checklist.

Case series

Author	Domain										Total
	1	2	3	4	5	6	7	8	9	10	
Abdullah, 2021	√	√	√	√		√	√	√	√	√	9
Afana, 2020	√	√	√	√		√	√	√	√	√	9
Almalla, 2019	√	√	√	√	√	√	√	√	√		9
Alushi, 2019	√	√	√	√	√	√	√	√	√	√	10
Badiye, 2016	√	√	√	√	√	√	√	√	√	√	10
Bansal, 2016	√	√	√	√	√	√	√	√	√	√	10
Barrionuevo-Sanchez, 2022	√	√	√	√	√	√	√	√	√	√	10
Bashline, 2022	√	√	√	√		√	√	√	√	√	9
Bernhardt, 2019	√	√	√	√	√	√	√	√	√	√	10
Bernhardt, 2021	√	√	√	√	√	√	√	√	√	√	10
Boll, 2019	√	√	√	√	√	√	√	√	√	√	10
Boshara, 2021	√	√	√	√	√	√	√	√	√	√	10
Brandao, 2021	√	√	√	√	√	√	√		√	√	9
Casassus, 2015	√	√	√	√	√	√	√	√	√	√	10
Cheng, 2019	√	√	√	√	√	√	√	√	√	√	10
Chieffo, 2020	√	√	√	√	√	√	√	√	√	√	10
David, 2019	√	√	√	√	√	√	√	√	√	√	10
Davidsen, 2019	√	√	√	√	√	√	√	√	√	√	10
Doersch, 2015	√	√	√	√	√	√	√	√	√	√	10
Fagot, 2020	√	√	√	√		√	√	√	√	√	9
Fahad, 2020	√	√	√	√	√	√	√	√	√		9
Garan, 2019	√	√	√	√	√	√	√	√	√	√	10
Gaudard, 2015	√	√	√	√	√	√	√	√	√	√	10
Hegelstad, 2020	√	√	√	√	√	√	√	√	√	√	10
Higgins, 2011	√	√	√	√	√	√	√	√	√	√	10
Hritani, 2019	√	√	√	√	√	√	√	√	√	√	10
Kaki, 2019	√	√	√	√	√	√	√	√	√	√	10
Kamran, 2020	√	√	√	√	√	√	√	√	√		9
Karatolios, 2020	√	√	√	√	√	√	√	√	√	√	10
Kennel, 2021	√	√	√	√	√	√	√	√	√	√	10
Lackermair, 2016	√	√	√	√	√	√	√	√	√	√	10
Lamarche, 2011	√	√	√	√	√	√	√	√	√	√	10
Lemaire, 2014	√	√	√	√	√	√	√	√	√	√	10
Maniuc, 2019	√	√	√	√	√	√	√	√	√	√	10
Manzo-Silberman, 2013	√	√	√	√	√	√	√	√	√	√	10
Meyns, 2003	√	√	√	√	√	√	√	√	√	√	10
Mierke, 2020	√	√	√	√	√	√	√	√	√	√	10
Nelson, 2021	√	√	√	√	√	√	√	√	√	√	10
Nouri, 2020	√	√	√	√	√	√	√	√	√	√	10
Ouweneel, 2019	√	√	√	√	√	√	√	√	√	√	10
Panoulas, 2021	√	√	√	√	√	√	√	√	√	√	10
Radakovic, 2021	√	√	√	√	√	√	√	√	√	√	10
Ramzy, 2021	√			√	√	√	√	√	√	√	8
Rock, 2022	√			√	√	√	√	√	√	√	8

Rohm, 2020	✓	✓	✓	✓	✓	✓	✓	✓	✓	✓	10
Schäfer, 2021	✓	✓	✓	✓	✓	✓	✓	✓	✓	✓	10
Schiller, 2016	✓	✓	✓	✓	✓	✓	✓	✓	✓	✓	10
Schroeter, 2016	✓	✓	✓	✓	✓	✓	✓	✓	✓	✓	10
Schurtz, 2020	✓	✓	✓	✓	✓	✓	✓	✓	✓	✓	10
Scolari, 2022	✓	✓	✓			✓	✓	✓	✓	✓	8
Sugimura, 2021	✓	✓	✓	✓	✓	✓	✓	✓	✓	✓	10
Takahashi, 2021	✓	✓	✓	✓	✓	✓	✓	✓	✓	✓	10
Tarabichi, 2020	✓	✓	✓	✓	✓	✓	✓	✓	✓	✓	10
Tepper, 2017	✓	✓	✓	✓	✓	✓	✓	✓	✓	✓	10
Trpkov, 2020	✓	✓	✓	✓		✓	✓	✓	✓	✓	9
Vase, 2017	✓	✓	✓	✓	✓	✓	✓	✓	✓	✓	10
Vasin, 2020	✓	✓	✓	✓	✓	✓	✓	✓	✓	✓	10
Wilkins, 2019	✓	✓	✓	✓	✓	✓	✓	✓	✓	✓	10
Zaiser, 2021	✓	✓	✓	✓	✓	✓	✓		✓	✓	9
Zubarevich, 2022	✓	✓	✓			✓	✓	✓	✓	✓	8

Domains:
1: Were there clear criteria for inclusion in the case series?
2: Was the condition measured in a standard, reliable way for all participants included in the case series?
3: Were valid methods used for identification of the condition for all participants included in the case series?
4: Did the case series have consecutive inclusion of participants?
5: Did the case series have complete inclusion of participants?
6: Was there clear reporting of the demographics of the participants in the study?
7: Was there clear reporting of clinical information of the participants?
8: Were the outcomes or follow up results of cases clearly reported?
9: Was there clear reporting of the presenting site(s)/clinic(s) demographic information?
10: Was statistical analysis appropriate?

<u>Cohort studies</u>

Author	Domain											Total
	1	2	3	4	5	6	7	8	9	10	11	
Au, 2021	✓		✓	✓	✓	✓	✓	✓	✓	✓	✓	10
Haurand, 2021	✓	✓	✓	✓	✓	✓	✓	✓	✓	✓	✓	11
Karatolios, 2021	✓	✓	✓	✓	✓	✓	✓	✓	✓	✓	✓	11
Nersesian, 2021	✓	✓	✓	✓	✓	✓	✓	✓	✓	✓	✓	11
Pappalardo, 2017	✓	✓	✓	✓	✓	✓	✓	✓	✓	✓	✓	11
Scherer, 2020	✓	✓	✓	✓	✓	✓	✓	✓	✓	✓	✓	11
Shibasaki, 2022	✓	✓	✓			✓	✓	✓	✓	✓	✓	9
Sieweke, 2021	✓	✓	✓	✓	✓	✓	✓	✓	✓	✓	✓	11
Syntila, 2021	✓	✓	✓	✓	✓	✓	✓	✓	✓	✓	✓	11

Domains:
1: Were the two groups similar and recruited from the same population?
2: Were the exposures measured similarly to assign people to both exposed and unexposed groups?
3: Was the exposure measured in a valid and reliable way?
4: Were confounding factors identified?
5: Were strategies to deal with confounding factors stated?
6: Were the groups/participants free of the outcome at the start of the study (or at the moment of exposure)?
7: Were the outcomes measured in a valid and reliable way?

8: Was the follow up time reported and sufficient to be long enough for outcomes to occur?
9: Was follow up complete, and if not, were the reasons to loss to follow up described and explored?
10: Were strategies to address incomplete follow up utilized?
11: Was appropriate statistical analysis used?

<u>Randomised controlled trials</u>

Author	Domain													Total
	1	2	3	4	5	6	7	8	9	10	11	12	13	
Ouweneel, 2017	✓	✓	✓				✓	✓	✓	✓	✓	✓	✓	9
Seyfarth, 2008	✓		✓				✓	✓	✓	✓	✓	✓	✓	8

1: Was true randomization used for assignment of participants to treatment groups?
2: Was allocation to treatment groups concealed?
3: Were treatment groups similar at the baseline?
4: Were participants blind to treatment assignment?
5: Were those delivering treatment blind to treatment assignment? □
6: Were outcomes assessors blind to treatment assignment?
7: Were treatment groups treated identically other than the intervention of interest?
8: Was follow up complete and if not, were differences between groups in terms of their follow up dequately described and analyzed?
9: Were participants analyzed in the groups to which they were randomized?
10: Were outcomes measured in the same way for treatment groups?
11: Were outcomes measured in a reliable way?
12: Was appropriate statistical analysis used?
13: Was the trial design appropriate, and any deviations from the standard RCT design (individual randomization, parallel groups) accounted for in the conduct and analysis of the trial?

Table S8. Grading of Recommendations, Assessment, Development, and Evaluations**.**

No. of studies	Certainty assessment						Effect			Certainty	Importance
	Study design	Risk of bias	Inconsistency	Indirectness	Imprecision	Other considerations	No. of events	No. of individuals	Rate (95% CI)		
Short-term Mortality (assessed with: %; Scale from: 0 to 100)											
63	observational studies	not serious [a]	not serious [b]	not serious [c]	not serious [d]	none	-	3896	46.5% (42.7-50.3%)	⨁⨁⨁⨁ HIGH	IMPORTANT
90-day Mortality (assessed with: %; Scale from: 0 to 100)											
5	observational studies	not serious [a]	not serious [e]	not serious [c]	not serious [f]	none	-	448	41.8% (34.4-49.3%)	⨁⨁⨁⨁ HIGH	IMPORTANT
6-month Mortality (assessed with: %; Scale from: 0 to 100)											
9	observational studies	not serious [a]	not serious [e]	not serious [c]	not serious [f]	none	-	676	51.1% (45.2-57.0%)	⨁⨁⨁⨁ HIGH	IMPORTANT
1-year Mortality (assessed with: %; Scale from: 0 to 100)											
10	observational studies	not serious [a]	not serious [e]	not serious [c]	not serious [f]	none	-	881	54.3% (48.9-59.7%)	⨁⨁⨁⨁ HIGH	IMPORTANT
Minor or Major Hemolysis (assessed with: %; Scale from: 0 to 100)											
32	observational studies	not serious [a]	not serious [g]	not serious [c]	not serious [h]	none	-	1708	24.9% (14.9-36.4%)	⨁⨁⨁⨁ HIGH	IMPORTANT
Access-site Bleeding (assessed with: %; Scale from: 0 to 100)											
23	observational studies	not serious [a]	serious [i]	not serious [c]	not serious [f]	none	-	1679	25.8% (14.7-38.5%)	⨁⨁⨁◯ MODERATE	IMPORTANT
Minor or Major Device Malfunction (assessed with: %; Scale from: 0 to 100)											

17	observational studies	not serious [a]	not serious	not serious [c]	not serious [f]	none	-	690	6.0% (3.1-9.3%)	⨁⨁⨁⨁ HIGH	IMPORTANT
Limb Ischemia (assessed with: %; Scale from: 0 to 100)											
32	observational studies	not serious [a]	not serious	not serious [c]	not serious [f]	none	-	**2351**	6.1% (3.7-8.9%)	⨁⨁⨁⨁ HIGH	IMPORTANT
Stroke (assessed with: %; Scale from: 0 to 100)											
32	observational studies	not serious [a]	not serious [e]	not serious [c]	not serious [f]	none	-	**1276**	5.5% (2.9-8.5%)	⨁⨁⨁⨁ HIGH	IMPORTANT

Explanations

a. Joanna Briggs Institute critical appraisal of articles found that the articles were of high quality
b. There is substantial heterogeneity, some of which was accounted for by meta-regression.
c. The study population in all studies were directly applicable to the population this systematic review was interested in.
d. The 95% CI and pooled estimate is within range to a previous study involving the US Nationwide Inpatient Sample (50.7% survival to discharge) and previous systematic review by Iannaccone, 2020 (47.8%; 95% 43.7-52%).
e. Heterogeneity is low (I^2 < 40%) to moderate (I^2 = 30-60%).
f. The 95% CI is not wide compared to the pooled estimate and does not affect clinical decision making.
g. Heterogeneity was considerable, but likely to be due to the high value reported by Badiye et al (62.5%)
h. Though the 95% CI is wide compared to the pooled estimate and higher than previously reported results, it is likely due to the high value reported by Badiye et al due to a difference in the definitions of hemolysis.
i. Heterogeneity was considerable.

References

1. Ouweneel DM, Eriksen E, Sjauw KD et al. Percutaneous Mechanical Circulatory Support Versus Intra-Aortic Balloon Pump in Cardiogenic Shock After Acute Myocardial Infarction. J Am Coll Cardiol 2017;69:278-287.

2. Ouweneel DM, de Brabander J, Karami M et al. Real-life use of left ventricular circulatory support with Impella in cardiogenic shock after acute myocardial infarction: 12 years AMC experience. Eur Heart J Acute Cardiovasc Care 2019;8:338-349.

3. Tepper S, Masood MF, Baltazar Garcia M et al. Left Ventricular Unloading by Impella Device Versus Surgical Vent During Extracorporeal Life Support. Ann Thorac Surg 2017;104:861-867.

4. Badiye AP, Hernandez GA, Novoa I, Chaparro SV. Incidence of hemolysis in patients with cardiogenic shock treated with impella percutaneous left ventricular assist device. ASAIO Journal 2016;62:11-14.

5. Casassus F, Corre J, Leroux L et al. The use of impella 2.5 in severe refractory cardiogenic shock complicating an acute myocardial infarction. Journal of Interventional Cardiology 2015;28:41-50.

6. Lang RM, Badano LP, Mor-Avi V et al. Recommendations for cardiac chamber quantification by echocardiography in adults: an update from the American Society of Echocardiography and the European Association of Cardiovascular Imaging. J Am Soc Echocardiogr 2015;28:1-39.e14.
